# Hair Metal Concentrations in Adults Living in Campania, Italy: A Cross-Sectional Biomonitoring Study in a Mixed Geogenic–Anthropogenic Exposure Setting

**DOI:** 10.3390/toxics14090768

**Published:** 2026-08-27

**Authors:** Rossella Farina, Juri Rimauro, Silvio Del Pizzo, Angelo Riccio, Elena Chianese

**Affiliations:** 1 Department of Science and Technology, Parthenope University of Naples, Centro Direzionale, Isola C4, 80143 Naples, Italy; rossella.farina001@studenti.uniparthenope.it (R.F.); silvio.delpizzo@uniparthenope.it (S.D.P.); elena.chianese@uniparthenope.it (E.C.); 2 Laboratorio Impatti sul Territorio e nei Paesi in Via di Sviluppo, ENEA Centro Ricerche Portici, P.le Enrico Fermi, 1, 80055 Portici, Italy; juri.rimauro@enea.it

**Keywords:** hair biomonitoring, trace elements, ICP-MS, lead, cadmium, chromium, Campania, human biomonitoring, scalp hair, toxic metals, environmental exposure

## Abstract

Human biomonitoring in environmentally complex regions requires not only matrices that are easy to collect but also careful consideration of external contamination, non-detects, and analytical uncertainty. Inductively coupled plasma mass spectrometry (ICP–MS) was used to measure the concentrations of 24 elements in occipital scalp-hair samples from 134 adults living in Campania, southern Italy (67 women and 67 men; age range: 20–72 yr). Continuous models were restricted to elements detected in at least 70% of adults and were fitted using HC3 heteroskedasticity-consistent standard errors, with Benjamini–Hochberg correction for the false discovery rate (FDR). Ca, Zn, Mg, K and Cu were the most abundant elements in the hair profile. The most consistent adjusted association was lower concentrations of Ca, Mg, Cu, Cr and Zn in men. Pb showed a strongly right-skewed distribution, whereas Cd was detected in only 22.4% of adults; this low detection frequency did not support a population-level elevation claim. Spatial summaries and clustering were retained for exploratory purposes: Kruskal–Wallis tests indicated a zone-level difference only for Zn, whereas patterns in Pb, Cr, As, Cd, and V were interpreted as priorities for confirmatory sampling rather than as evidence of territorial risk. Sensitivity analyses based on the limit of detection (LOD) supported retaining As and Cd as descriptive outcomes; V associations were not promoted to primary findings because the V detection frequency was below the pre-specified threshold. These findings support the use of hair biomonitoring as a non-invasive screening approach in mixed geogenic–anthropogenic settings, provided that sex, sample mass, non-detects, outliers, and external contamination are considered explicitly and that confirmatory matrices are used where appropriate.

## 1. Introduction

Metals and metalloids remain among the most persistent environmental stressors because they can be redistributed through air, soil, water, and food chains and may exert toxic effects at low chronic doses, depending on their chemical form, target organ, and the life stage at exposure. Evidence linking Pb, Cd, As and other elements to neurodevelopmental, renal, cardiovascular and carcinogenic outcomes is well established [1,2,3,4]. At the same time, several elements measured in multi-element panels, including Cu, Zn, Se, Mg and Ca, are essential nutrients whose interpretation differs fundamentally from that of non-essential toxicants. This dual toxicological and nutritional role is one reason why multi-element biomonitoring studies should avoid the simplistic assumption that “high equals harmful” and should instead focus on distributions, determinants, analytical robustness, and follow-up hypotheses.

Contemporary human biomonitoring has moved from single-substance surveillance toward harmonised, multi-chemical frameworks that integrate exposure assessment, quality assurance and policy interpretation. The European Human Biomonitoring Initiative (HBM4EU) aimed to harmonise sampling, analytical performance, and interpretation across countries and developed guidance values for selected substances for which robust toxicological and epidemiological evidence exists [5,6,7,8]. These developments are important for the present work for two reasons. First, they highlight the need for transparent pre-analytical and analytical procedures when comparing biomonitoring results across studies. Second, they show that not every biological matrix or element has an established health-based interpretive framework. For many metals, blood or urine remain the preferred matrices for quantitative exposure assessment, whereas hair is better viewed as a complementary, time-integrated and hypothesis-generating biospecimen [4,9].

**Figure 1:**
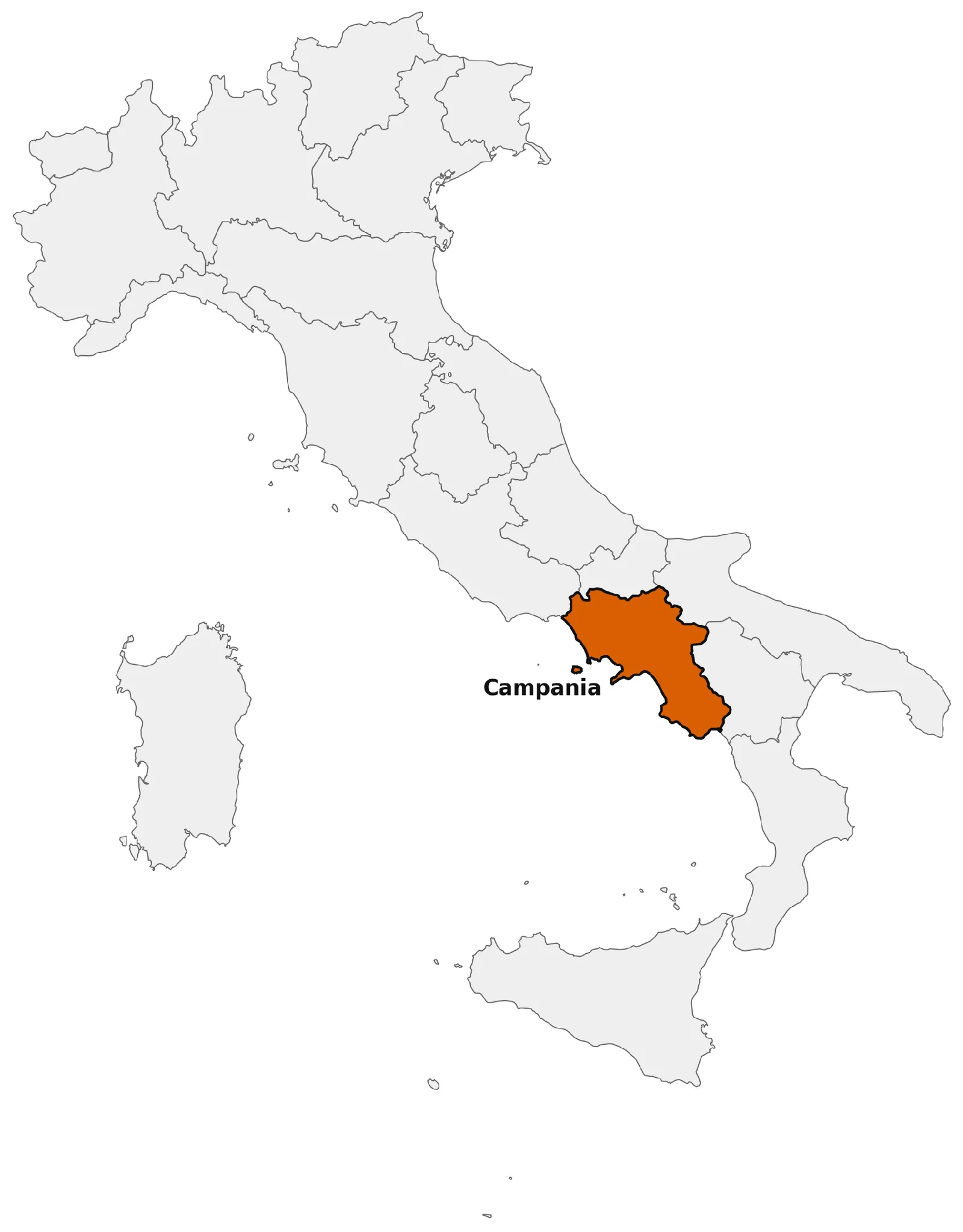
Study location. Campania is highlighted in orange on the map of Italy. The map is provided for geographic context and does not represent participant density or sampling intensity.

Campania (see Figure 1) is a useful setting for human biomonitoring because geogenic and anthropogenic drivers coexist. Volcanic bedrock, dense traffic, industrial activities and historical illegal dumping in parts of Naples and Caserta create a mixed-exposure landscape where environmental measurements alone do not necessarily reflect internal burden [10,11,12,13]. The “Land of Fires” debate has also shown how difficult it is to translate environmental contamination into individual exposure without well-designed biomonitoring and exposure reconstruction [14]. In this context, hair analysis can complement soil geochemistry, atmospheric dispersion modelling, and air-quality post-processing by integrating exposure across multiple pathways at the individual level [15,16,17,18].

Scalp hair is attractive because collection is non-invasive, storage is simple, and many elements occur at higher concentrations than in blood or urine [9,19,20,21]. Proximal hair segments can provide a short retrospective exposure window, which is useful when repeated blood or urine collection is not feasible. However, interpretation is difficult. External contamination from dust, traffic particles, water, and cosmetic products can persist despite washing; elemental incorporation into hair varies with sex, melanin content, nutritional status, and metabolic factors; and published reference intervals differ widely across washing protocols, digestion procedures, instrumentation, and cohort composition [20,22,23,24]. Recent studies using spatially resolved laser-ablation ICP–MS reinforce the conclusion that external contamination remains a central unresolved challenge in elemental hair analysis, while evaluations of washing procedures show that pre-cleaning can alter results without fully resolving this problem [21,24,25,26]. For these reasons, hair concentrations should be treated as biomarkers of elemental burden and potential contamination, not as direct dose estimates.

The objective of this study was to provide a cautious adult biomonitoring profile for Campania by (i) describing the detection frequencies and distributions of 24 elements in hair; (ii) testing associations with sex, age, lifestyle, and questionnaire variables; (iii) evaluating outliers, sample mass, and the handling of non-detects; (iv) exploring territorial patterns for selected sentinel elements; and (v) comparing median concentrations in Campanian adults with those reported in Italian studies, without treating these comparisons as formal reference benchmarks [27,28,29]. Reporting follows STROBE recommendations for cross-sectional observational studies [30].

## 2. Materials and Methods

### 2.1. Study Design and Population

We conducted a cross-sectional biomonitoring study among residents of Campania. The original dataset included 148 participants. Fourteen individuals younger than 20 yr were excluded before analysis because this work was designed as an adult biomonitoring study and did not include a paediatric sampling protocol. The final analytic cohort therefore comprised 134 adults aged 20–72 yr, balanced by sex (67 women and 67 men). For details, see Table 1. All participants provided written informed consent before sample collection.

### 2.2. Hair Sampling and Preparation

Hair samples comprising the proximal 3–4 cm were collected from the occipital scalp with stainless-steel scissors. Samples were sequentially washed in acetone, twice in ultrapure water, and again in acetone, with ultrasonic agitation for 20 min at each step. They were then dried at 40 °C for 24 h before weighing. Approximately 150 mg of dried hair was digested in 3 mL of nitric acid for 24 h, after which 0.5 mL of hydrogen peroxide was added and digestion continued for a further 24 h. The final digests were diluted to 25 mL with ultrapure water and filtered through a 0.2 μm membrane to remove residual solids that might interfere with ICP–MS analysis. This protocol was intended to reduce external contamination while preserving comparability with previous hair-biomonitoring studies [19,20,31].

### 2.3. Analytical Determination and Quality Assurance

Concentrations of Ag, Al, As, B, Ba, Be, Bi, Ca, Cd, Co, Cr, Cu, Fe, K, Mg, Mn, Mo, Ni, Pb, Se, Tl, U, V and Zn were quantified using an Agilent 7850 ICP–MS system (Agilent Technologies, Santa Clara, CA, USA). Multi-element internal standards were used to correct for instrumental drift and matrix effects, and results were expressed as μg/g dry weight.

To improve reproducibility, the Supplementary Material was expanded to document the analytical platform and the available calibration and quality-control information needed for an ICP–MS audit trail. Laboratory limits of detection (LODs) and quantification (LOQs) were reported for 20 elements, including As, Cd and V, and incorporated into the sensitivity analyses for low-detection elements. The archived dataset contained final concentration data, hair-mass measurements and questionnaire variables. Quality-assurance (QA) procedures included procedural blanks and replicate digestions of a subset of samples. However, neither a matrix-matched certified reference material for hair nor a controlled sample-mass gradient experiment was available for this dataset. Extreme values for Pb, Cd, Se and Ag were flagged for targeted review of sample identity and any available batch, blank and recovery records, as well as for possible exogenous contamination. Because the washing protocol can reduce but not eliminate external contamination, and because validation of hair as an analytical matrix remains challenging [32,33], the analysis emphasises distributional patterns, sensitivity analyses and follow-up needs rather than direct risk quantification.

### 2.4. Statistical Analysis

Values below the detection limit were coded as zero for data management and handled using the offset-based approach described below. This pragmatic approach was adopted because of the sample size and the study’s primary focus on elements with ≥70% detection; formal censored-data methods would be preferable in larger studies (see Section 4.1). Descriptive statistics included detection frequency, median, interquartile range, maximum, and the geometric mean of positive observations (GM^+^). Arithmetic means were reported only in the Supplementary Material because right-skewed distributions make means poor summaries for several elements.

Inferential models were restricted to elements detected in at least 70% of adults. In the adult-only cohort, these were Al, Ba, Ca, Cr, Cu, K, Mg, Pb, Se and Zn. Fe was marginally below the threshold (69.4%) and was not modelled continuously. For each primary element, the outcome was log-transformed after adding an element-specific offset equal to half the minimum positive value observed among adults. Expanded multivariable ordinary least-squares models were fitted using HC3 heteroskedasticity-consistent standard errors. Predictors were male sex; fish consumption (1–3 times/week and >3 times/week, with <1 time/week as the reference); current smoking; passive smoke exposure; hair treatments; cosmetic product use; tap water use; medication use; supplement use; occupational/environmental risk; age; and sample mass. The occupational/environmental-risk variable was coded from the questionnaire item asking whether participants perceived their living or working environment as being at risk; it should therefore be interpreted as a broad self-reported screening indicator rather than a measured exposure variable. Effect sizes were expressed as percentage changes, $100\times [\exp\left(\hat{\beta }\right)-1]$. FDR correction used the Benjamini–Hochberg method within each predictor family across the ten primary elements. Global FDR values across all regression coefficients are provided in the Supplementary Material. To assess robustness for low-detection elements, additional exploratory analyses were performed for As, Cd and V using three approaches: zero-coded values with an LOD-based log offset, substitution of non-detects with one-half of the laboratory LOD, and left-censored Tobit regression using the laboratory LOD as the censoring point. These models were treated as robustness checks rather than as primary inferential analyses.

Median quantile regression using the same covariate set was performed as a sensitivity analysis. For low-detection elements, associations between detection status and binary predictors were evaluated using Fisher’s exact tests. Spearman correlations among primary elements were computed on log-transformed shifted concentrations, with FDR correction across element pairs.

#### 2.4.1. Exploratory Spatial Aggregation

The spatial analysis was designed to provide a descriptive link between individual-level hair biomonitoring and the geography of the study area. Municipalities of residence were grouped into four contiguous macro-areas with broadly comparable territorial features: Naples, North Naples, South Naples and the Sorrento Peninsula. This aggregation reduced visual fragmentation in the maps and allowed the main spatial patterns to be examined without disclosing municipality-level identifiers.

The territorial display focused on nine elements selected a priori to facilitate interpretation. Pb, Cr, As and Cd were mapped because of their toxicological relevance; Al, Fe, Mn and V were included as potential sentinels of crustal, particulate, or industrial sources; and Zn was included because it was abundant in hair and belonged to the main mineral profile. Adults living outside the four predefined mapping groups were excluded from this visualization, leaving 109 participants (Naples, n=32; North Naples, n=38; South Naples, n=30; Sorrento Peninsula, n=9). To limit the influence of extreme observations, zone-level medians were used for Al, Cr, Fe, Mn, Pb and Zn. Because detections of As, Cd and V were infrequent, these elements were mapped by detection frequency rather than by arithmetic mean. Exploratory Kruskal–Wallis tests were used to compare concentrations across zones, followed by pairwise Mann–Whitney tests with Benjamini–Hochberg correction when the global test suggested heterogeneity. For As, Cd and V, zone-level detection frequencies were compared using contingency-table tests. Because the Sorrento Peninsula stratum contained only nine adults, bootstrap percentile intervals for the median were also calculated to assess the stability of the mapped values. The Sorrento Peninsula stratum was treated as precision-limited because only nine adults contributed data; its summaries were therefore interpreted as unstable descriptive estimates rather than as a basis for territorial ranking.

#### 2.4.2. Exploratory Clustering Approach

Unsupervised clustering was performed as an exploratory, phenotype-oriented analysis rather than as source apportionment. The input matrix included the same ten primary elements used for continuous modelling. Concentrations were transformed as $\log(x+o_{e})$, where $o_{e}$ was half the minimum positive adult value for element *e*. The transformed values were then winsorised at the 1st and 99th percentiles and robustly standardised using the median and interquartile range. Ward’s hierarchical clustering method was selected as the primary approach because it is transparent and minimises within-cluster dispersion in Euclidean space [34]. Candidate solutions with k=2 to k=5 were compared using average silhouette width [35], minimum cluster size, and bootstrap stability assessed with the adjusted Rand index. Gaussian mixture models with diagonal and full covariance matrices were fitted as a sensitivity analysis and compared using the Bayesian information criterion [36]. To evaluate whether clustering mainly reflected known covariates, a residualised analysis was also performed after regressing each transformed element on sex, age, hair treatments, cosmetic product use, and sample mass. Element-level clustering was performed on a distance matrix defined as 1−|ρ|, where ρ is the Spearman correlation coefficient.

## 3. Results

### 3.1. Detection Frequencies and Descriptive Statistics

Table 2 summarises detection frequencies and distributions. Ten elements met the 70% detection criterion: Al, Ba, Ca, Cr, Cu, K, Mg, Pb, Se and Zn. Fe was close to the threshold but remained below it. Ca, Zn, Mg, K and Cu were the most abundant elements by mass. Pb was characterised by a moderate median but a very long right tail, and Cd was detected in only 22.4% of adults.

### 3.2. Exploratory Spatial Distribution of Sentinel Elements

**Figure 2:**
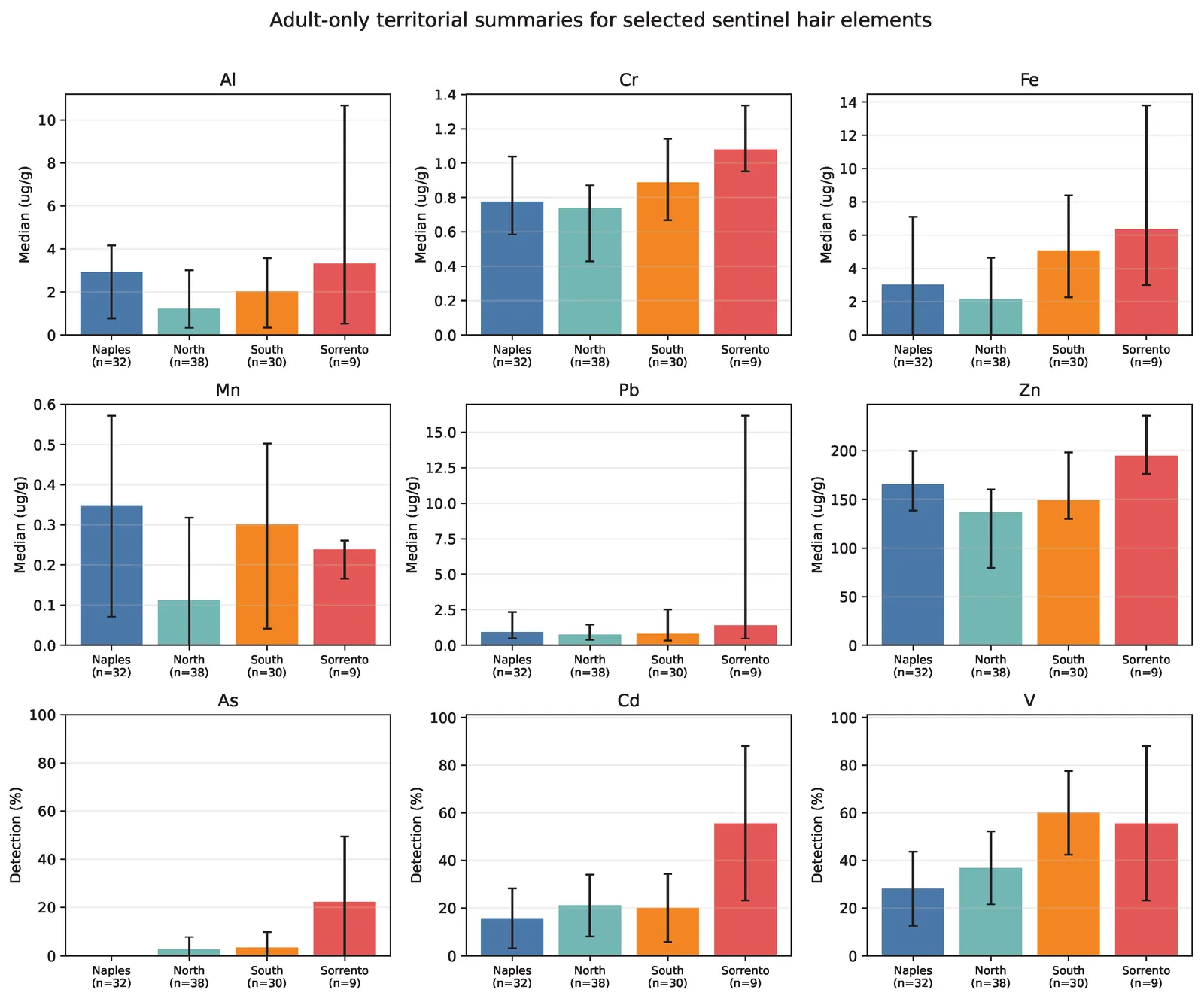
Exploratory adult-only territorial summaries for selected sentinel elements. Concentration panels (Al, Cr, Fe, Mn, Pb and Zn) show zone medians with interquartile-range error bars; detection panels (As, Cd and V) show detection percentages with approximate 95% binomial confidence intervals. Group sizes were Naples (n=32), North Naples (n=38), South Naples (n=30), and the Sorrento Peninsula (n=9). The small Sorrento Peninsula stratum limits precision, and all panels are intended for descriptive, hypothesis-generating interpretation rather than territorial risk ranking.

Figure 2 summarises nine selected elements across the four territorial groups. The bar-chart panels improve readability and display variability. Error bars represent interquartile ranges for concentration panels and approximate 95% binomial confidence intervals for detection-frequency panels. The figure places the biomonitoring results in a geographical context while making the principal limitation of the analysis explicit: the mapped values are descriptive summaries from a convenience sample of adults and should not be interpreted as estimates of territorial risk. Estimation precision is particularly limited for the Sorrento Peninsula (n=9). Its medians and detection frequencies should therefore be read as uncertain screening summaries rather than stable area-level estimates. A confirmatory spatial study should use stratified recruitment with a larger Sorrento Peninsula stratum to reduce sampling imbalance and improve precision.

Global Kruskal–Wallis tests did not indicate overall spatial differences for Al, Mn or Pb (all p>0.20), whereas Cr and Fe showed only borderline evidence of heterogeneity (p=0.062 and p=0.077, respectively). Zn was the only mapped concentration element with a nominal global difference across zones (H=9.11, p=0.028); after pairwise correction, the contrast between North Naples and the Sorrento Peninsula remained significant (q=0.040). The Sorrento Peninsula had the highest medians for Cr (1.08 μg/g, Fe (6.35 μg/g), Pb (1.41 μg/g) and Zn (194.98 μg/g), but bootstrap intervals were wide for several elements because this stratum included only nine adults. Among the low-detection elements, As detection frequency differed across zones (p=0.018), whereas Cd and V showed borderline evidence of heterogeneity (p=0.052 and p=0.055, respectively). These patterns highlight priorities for confirmatory sampling but do not establish robust area-level differences in exposure.

### 3.3. Expanded Multivariable Regression Models

Sex was the predictor with the strongest and most reproducible adjusted associations. Men had lower concentrations of Ca, Mg, Cu, Cr and Zn after FDR correction (Table 3). The direction of these effects was consistent in median quantile regression (Supplementary Table S3). No association with fish consumption, medication use, supplement use, current smoking, passive smoke exposure, tap water use, or occupational/environmental risk survived FDR correction. Sample mass was inversely associated with Mg and Zn in the HC3 models and with several elements in quantile regression (Supplementary Table S3).

### 3.4. Detection-Probability Patterns Among Low-Detection Elements

For elements below the 70% detection threshold, Fisher’s exact tests indicated sex-related differences in the detection of Ag, Co, Mn, Ni and V, with detection frequencies generally higher among women. Hair treatments were associated with detection of Co, Mn, Ni and V. Additional sensitivity analyses for As, Cd and V used the available laboratory LOD values and compared zero-offset models, LOD/2 substitution, and LOD-based Tobit modelling (Supplementary Tables S16 and S17). These analyses did not alter the main conclusion that As and Cd should not be evaluated using standard continuous regression in this dataset. V retained FDR-significant associations with sex and age only in the Tobit sensitivity model; this finding was not treated as a primary result because V remained below the 70% detection threshold.

### 3.5. Inter-Element Correlations

Spearman correlations among the ten primary elements showed a coherent mineral cluster involving Ca, Mg, Cu and Zn; Cr was also positively correlated with several elements. Pb correlated positively with Ba and several other elements. The full correlation matrix is shown in Table 4.

### 3.6. Exploratory Clustering

Hierarchical clustering of the ten primary elements provided clearer support for a simple two-profile description than for a high-dimensional exposure classification. In the raw concentration space, the k=2 solution had an average silhouette width of 0.377 and separated 105 adults with generally higher mineral concentrations from 29 adults with lower concentrations across Ca, Mg, Cu, Zn and several trace elements (Supplementary Tables S6 and S7. The larger cluster had higher median concentrations of Ca (1410.0 vs. 640.3 μg/g), Mg (117.5 vs. 41.5 μg/g), Cu (16.5 vs. 8.8 μg/g) and Zn (161.3 vs. 99.4 μg/g). The smaller cluster had a higher proportion of men (65.5% vs. 45.7%) and lower median sample mass, but the demographic and questionnaire associations were weak and should be regarded as descriptive.

A three-cluster sensitivity analysis produced a similar silhouette width (0.378) but included a very small four-participant subgroup. This subgroup had low sample mass, a high prevalence of self-reported occupational/environmental risk, and markedly higher median Pb and Ba concentrations than the other clusters (Supplementary Table S8). Because the subgroup was small and included observations from the extreme right tail of the Pb distribution, it is best interpreted as a sentinel pattern requiring QA and follow-up rather than as an independently validated exposure class. Residual clustering after adjustment for sex, age, hair treatments, cosmetic products and sample mass was less structured: the k=2 residual solution had a silhouette width of 0.183 and no strong association with the recorded covariates (Supplementary Table S9). Gaussian mixture models were also unstable across preprocessing choices: BIC preferred a four-component full-covariance model for raw profiles but a one-component full-covariance model for residual profiles (Supplementary Table S10). The statistical diagnostics therefore indicated stronger support for broad profile differences than for stable, discrete exposure classes.

### 3.7. Contextual Comparison with Italian Populations

Compared with Palermo schoolchildren and adults from the Domizio Flegreo–Agro Aversano district, Campanian adults in this study had broadly comparable median Pb concentrations but a higher median Cr concentration (0.80 μg/g) than the published Italian comparators [27,29]. The Palermo study provides a southern Italian, urban, hair-based comparator, but not a strict benchmark: its cohort was paediatric, the physiological and behavioural determinants of hair composition differ between children and adults, and the analytical protocols were not identical. The Domizio study is geographically closer and included adults, but it represents a specific sub-area with documented geochemical characterisation of soil, groundwater, crops, and hair, including Pb isotope data [29]. Because cohorts differ in age, geography, washing protocol and instrumentation, these comparisons are descriptive only. Cd cannot be meaningfully compared on the basis of median concentrations because most values in the adult cohort were below the detection limit.

## 4. Discussion

This adult-only analysis brings together three perspectives that are often discussed separately: metal exposure in environmental health, European biomonitoring practice, and the methodological limits of hair analysis. The clearest reproducible result was the association of sex with several hair elements, particularly Ca, Mg, Cu, Cr and Zn. This pattern is consistent with prior literature reporting sex differences in hair elemental composition and with the known influence of hormonal, nutritional, morphological and cosmetic factors on hair mineral profiles [37,38,39]. Importantly, the finding is not limited to a single element but appears as a coordinated shift within a correlated mineral cluster. This coordinated pattern is less likely to reflect chance alone, although residual confounding by hair care, diet, and unmeasured biological factors remains possible.

The absence of robust associations with fish consumption, medication use, supplement use, current smoking, passive smoke exposure, tap water use, and occupational/environmental risk should be interpreted carefully. It does not prove that these pathways are irrelevant in Campania. Rather, it suggests that the available questionnaire variables were too broad, too imbalanced, or too imprecisely measured to explain inter-individual variation after FDR correction. For example, fish consumption was recorded by frequency category but not by species, source, portion size, or local origin; tap water use did not capture plumbing materials or household water chemistry; and occupational/environmental risk was necessarily self-reported. A future source-oriented protocol should therefore replace broad indicators with exposure-specific modules and environmental co-samples.

Element-specific interpretation must remain cautious because concentrations in hair cannot be translated directly into health-based risk estimates without confirmatory matrices [4,40]. The following discussion therefore treats Pb, Cd and Cr as screening signals rather than as direct evidence of internal dose or toxicity.

The strongly right-skewed Pb distribution warrants particular attention. The adult median was modest, but the maximum value exceeded 887 μg/g, making the arithmetic mean misleading. Such an extreme value should not be used to infer population-level Pb burden without confirmatory QA. It may reflect a genuinely high level of individual exposure, a surface-contamination event, occupational or residential exposure, or an analytical or transcription error. A targeted audit and, if possible, follow-up involving blood Pb measurement, household dust and tap water testing, and a detailed residential and occupational history would be appropriate. Because blood Pb is a better-established biomarker of recent internal exposure than hair Pb, any public-health interpretation of the outlier should be based on confirmatory sampling rather than on hair alone [4,40].

Cd was detected in only 22.4% of adults; therefore, the dataset does not support a claim of elevated population-level Cd exposure. This is an important negative result because Cd is a priority substance in European biomonitoring and HBM4EU guidance-value work, especially for renal effects [7,8]. However, urine Cd, not hair Cd, is generally more appropriate for long-term Cd body burden. The current hair data should therefore motivate confirmatory urine-based assessment only if independent evidence suggests possible Cd exposure.

Cr was detected in most adults, and its median concentration was higher than those reported for the contextual Italian comparators. This signal is potentially important but requires caution because total hair Cr cannot distinguish essential trivalent Cr from other species, and source attribution requires environmental sampling, speciation or source-specific evidence. The result should be framed as a regionally relevant screening signal in a mixed-exposure setting rather than as evidence of health risk. If the Cr pattern is prioritised for follow-up, the next step should combine repeated hair analysis with urine or blood biomarkers where appropriate, local environmental sampling, occupational history and analytical checks for ICP–MS interferences.

The inverse association of sample mass with Mg and Zn should prompt quality-control scrutiny rather than biological interpretation. Because concentrations are already normalised by hair mass, a residual association with mass may reflect LOD propagation, weighing uncertainty, heterogeneous surface contamination, batch effects, or model instability at low sample weights. The present dataset did not include a serial sample-mass gradient experiment, so these mechanisms cannot be distinguished. The finding is therefore reported as an analytical signal that should be addressed in future work through matrix-matched certified reference materials for hair, element-specific LOD/LOQ reporting, spike-recovery assessment, replicate RSDs, and controlled mass-gradient tests.

The territorial display adds useful geographic context, but it should not be read as evidence of a regional exposure gradient. Apart from Zn, concentration differences were not statistically robust across zones. In particular, the Sorrento Peninsula estimate is based on only nine adults, which compromises precision and increases susceptibility to sampling imbalance. The spatial results are therefore most useful for planning confirmatory, stratified sampling rather than for ranking territories.

The clustering analysis adds nuance but does not change the main interpretation. Its strongest signal was a broad mineral-rich versus mineral-poor contrast, which is consistent with the regression finding that sex is a major determinant of Ca, Mg, Cu and Zn. The small high-Pb/high-Ba subgroup identified only in the three-cluster raw solution is potentially useful for targeted QA and source follow-up, but the weak residual clustering argues against presenting the clusters as stable exposure categories. This distinction is important: clustering can help prioritise samples and generate hypotheses, but it cannot disentangle endogenous incorporation, external deposition, hair-care effects, and analytical features without independent environmental or biological validation.

From a public-health perspective, the most defensible message is not that Campanian adults have a demonstrably elevated toxic-metal burden, but that adult hair biomonitoring can identify interpretable population patterns and sentinel individuals in a region where environmental sources are complex. The findings justify targeted follow-up for Pb outliers and Cr, stronger analytical harmonisation, and integration with environmental matrices. They do not justify hazard quotients, lifetime cancer-risk calculations or causal source attribution from hair data alone.

### 4.1. Limitations

Hair is vulnerable to external contamination and cosmetic effects even after washing. Two critical inherent weaknesses of this retrospective dataset are the absence of a matrix-matched certified reference material (CRM) for hair and the absence of a controlled sample-weight gradient validation experiment. These missing experimental conditions prevent retrospective assessment of matrix-specific analytical accuracy and of whether reported concentrations remain stable across the routine sample-mass range. Although laboratory LOD/LOQ values were incorporated into low-detection sensitivity analyses, the statistical archive also lacked complete whole-process spike-recovery documentation and complete instrument operating records. No blood, urine, or toenail samples; environmental co-samples; Pb isotope data; or Cr speciation data were available. The territorial analysis used four broad aggregations and excluded adults outside the predefined mapping groups; the Sorrento Peninsula stratum was especially small (n=9), limiting precision and increasing vulnerability to sampling bias. The clustering analysis was exploratory, sensitive to preprocessing and outliers, and intentionally interpreted as descriptive profiling rather than causal source apportionment. The questionnaire contained useful screening variables but lacked detailed exposure reconstruction for diet, occupation, residential history, indoor dust, drinking-water plumbing and product use. Finally, because this is a convenience adult cohort rather than a probability sample, it should not be used to derive regional reference intervals.

### 4.2. Future Directions

Future validation should be planned prospectively and embedded in each analytical batch. A practical workflow would include: (i) a sample-weight gradient using replicate aliquots from a homogenised pooled-hair material, with at least three to four masses spanning the routine working range and evaluation of concentration stability across masses; (ii) parallel digestion of a matrix-matched hair CRM alongside study samples, procedural blanks and replicate digestions, with recovery assessed against the certified values; and (iii) whole-process spike-recovery samples fortified before digestion so that recovery reflects digestion and instrumental measurement rather than the instrumental step alone. Acceptance criteria for recovery and precision should be defined before analysis, and batch-level recovery, blank and replicate records should be retained in the study archive. The confirmatory epidemiologic design should also use stratified recruitment by sex, age and territorial zone, with deliberate enlargement of the Sorrento Peninsula stratum to reduce sampling bias and narrow uncertainty around zone-specific summaries. Follow-up for Pb should include blood Pb measurements and Pb isotope or other source-tracing information where feasible; follow-up for Cd should prioritise urine Cd; and follow-up for Cr should address analytical interferences and, if possible, include speciation or complementary biomarkers. If minors are to be studied, they should be handled in a separate paediatric protocol with guardian consent and formal ethical review or documented exemption.

## 5. Conclusions

This adult-only cross-sectional study documents the concentrations of 24 elements in hair samples from 134 Campanian residents aged 20–72 yr. The most robust finding is lower concentrations of Ca, Mg, Cu, Cr and Zn in men after multivariable adjustment and FDR correction. Pb shows a strongly right-skewed distribution that requires individual-level QA and possible follow-up, whereas Cd was largely undetected and does not support a population-level elevation claim. Spatial tests and stability checks showed that territorial patterns are useful for identifying follow-up priorities but do not establish area-level risk gradients. Sensitivity analyses for low-detection elements indicated that As, Cd and V require detection-based or censored-data approaches rather than routine continuous modelling. Exploratory clustering identified broad differences in mineral profiles and a small potential Pb/Ba sentinel subgroup, but residual clustering was weak and did not support discrete source classes. Hair biomonitoring is useful as a screening and hypothesis-generating tool in Campania, but source attribution and risk assessment require confirmatory biomarkers, validated analytical quality assurance, and environmental co-sampling. 

## Figures and Tables

**Table 1:** Participant characteristics in the adult analytic cohort (n=134). Percentages use the full cohort as the denominator. Fish-consumption information was available for 131 participants; the displayed percentages retain n=134 as the denominator to remain consistent with the source questionnaire summary.

Characteristic	*n* or Summary	Percent (%)
Participants, *n*	134	100.0
Female sex	67	50.0
Male sex	67	50.0
Age, mean (range), yr	35.2 (20–72)	
Current smoker	40	29.9
Passive smoke exposure	54	40.3
Hair treatments	31	23.1
Cosmetic product use	27	20.1
Tap water use	65	48.5
Medication use	37	27.6
Supplement use	57	42.5
Occupational/environmental risk	53	39.6
Fish consumption: <1 time/week	34	25.4
Fish consumption: 1–3 times/week	84	62.7
Fish consumption: >3 times/week	13	9.7

**Table 2:** Detection frequency and concentration distributions in hair from Campanian adults (n=134). All concentration summaries are reported in μg/g dry weight. GM^+^ denotes the geometric mean among positive observations only. Elements marked * had detection frequencies below 70% and were excluded from the primary continuous regression models.

Element	Detected (%)	Median	P25	P75	Max	GM^+^
Ag *	55.2	0.164	0.000	0.608	75.89	0.671
Al	85.1	1.56	0.455	3.80	37.65	1.99
As *	3.7	0.000	0.000	0.000	1.56	0.325
B *	36.6	0.000	0.000	0.720	74.30	1.20
Ba	70.1	0.663	0.000	1.45	11.77	1.12
Be *	1.5	0.000	0.000	0.000	1.12	0.304
Bi *	3.0	0.000	0.000	0.000	0.893	0.322
Ca	99.3	1279.7	627.0	2667.2	9614.6	1258.8
Cd *	22.4	0.000	0.000	0.000	311.2	1.68
Co *	17.2	0.000	0.000	0.000	0.893	0.199
Cr	96.3	0.804	0.599	1.06	10.49	0.791
Cu	99.3	13.94	10.96	20.46	81.75	15.07
Fe *	69.4	3.07	0.000	6.98	92.96	5.10
K	97.0	14.79	4.95	33.90	311.4	14.16
Mg	99.3	101.7	41.57	191.6	763.5	90.41
Mn *	65.7	0.192	0.000	0.443	4.71	0.385
Mo *	23.1	0.000	0.000	0.000	18.36	0.340
Ni *	36.6	0.000	0.000	0.171	3.91	0.299
Pb	93.3	0.808	0.335	2.27	887.1	1.27
Se	85.1	0.562	0.282	0.835	106.3	0.630
Tl *	0.0	0.000	0.000	0.000	0.000	0.000
U *	9.0	0.000	0.000	0.000	0.530	0.220
V *	39.6	0.000	0.000	0.166	0.503	0.193
Zn	99.3	153.5	123.8	194.7	1008.1	147.7

**Table 3:** Adjusted associations meeting predictor-wise FDR q<0.10 in the adult-only HC3-robust log-linear models. $\hat{\beta }$ is the regression coefficient on the log scale, and $\Delta \%=100[\exp\left(\hat{\beta }\right)-1]$. Full model output is reported in Supplementary Table S2.

Predictor	Element	$\hat{\beta }$	95% CI (log)	Δ%	95% CI (%)	*p*	*q*
Male sex	Ca	−0.957	[−1.313, −0.601]	−61.6	[−73.1, −45.2]	1.35 × 10^−7^	1.35 × 10^−6^
Male sex	Cr	−0.401	[−0.684, −0.118]	−33.0	[−49.5, −11.2]	0.00544	0.0115
Male sex	Cu	−0.473	[−0.753, −0.192]	−37.7	[−52.9, −17.5]	0.000956	0.00319
Male sex	Mg	−0.738	[−1.121, −0.356]	−52.2	[−67.4, −29.9]	0.000154	0.000769
Male sex	Zn	−0.274	[−0.468, −0.080]	−23.9	[−37.4, −7.6]	0.00573	0.0115
Sample mass, per g	Mg	−3.035	[−4.547, −1.524]	−95.2	[−98.9, −78.2]	8.3 × 10^−5^	0.000415
Sample mass, per g	Zn	−2.638	[−3.441, −1.836]	−92.9	[−96.8, −84.1]	1.15 × 10^−10^	1.15 × 10^−9^

**Table 4:** Spearman correlation matrix for the ten primary elements after the study-specific log-shift transformation (n=134). Asterisks denote pairwise associations that remained significant after Benjamini–Hochberg correction (q<0.05).

	Al	Ba	Ca	Cr	Cu	K	Mg	Pb	Se	Zn
Al	1.00	0.37 *	0.32 *	0.53 *	0.42 *	0.35 *	0.38 *	0.46 *	0.35 *	0.35 *
Ba	0.37 *	1.00	0.31 *	0.16	0.30 *	0.68 *	0.46 *	0.55 *	0.34 *	0.40 *
Ca	0.32 *	0.31 *	1.00	0.32 *	0.61 *	0.12	0.76 *	0.25 *	0.07	0.41 *
Cr	0.53 *	0.16	0.32 *	1.00	0.45 *	0.33 *	0.35 *	0.34 *	0.50 *	0.49 *
Cu	0.42 *	0.30 *	0.61 *	0.45 *	1.00	0.22 *	0.50 *	0.36 *	0.34 *	0.48 *
K	0.35 *	0.68 *	0.12	0.33 *	0.22 *	1.00	0.45 *	0.50 *	0.41 *	0.48 *
Mg	0.38 *	0.46 *	0.76 *	0.35 *	0.50 *	0.45 *	1.00	0.39 *	0.21 *	0.56 *
Pb	0.46 *	0.55 *	0.25 *	0.34 *	0.36 *	0.50 *	0.39 *	1.00	0.31 *	0.37 *
Se	0.35 *	0.34 *	0.07	0.50 *	0.34 *	0.41 *	0.21 *	0.31 *	1.00	0.51 *
Zn	0.35 *	0.40 *	0.41 *	0.49 *	0.48 *	0.48 *	0.56 *	0.37 *	0.51 *	1.00

## Data Availability

De-identified data supporting the reported results are available from the corresponding author upon reasonable request, subject to privacy restrictions related to age, residence and questionnaire variables. Municipality-level identifiers are not publicly released to reduce re-identification risk in small territorial strata.

## Supplementary Materials

The following supporting information can be downloaded at https://www.mdpi.com/article/10.3390/toxics14090768/s1, Supplementary Tables S1–S18, the territorial summary and sensitivity analysis tables generated for Figure 2. Table S1. Arithmetic means of hair element concentrations in Campanian adults (*n* = 134). Units: µg/g dry weight. Means are provided for completeness but are not primary summaries for right-skewed elements. Table S2. Full HC3-robust multivariable regression output for the ten primary adult elements. Outcomes are log-transformed concentrations with an element-specific offset. Percentage changes are $100\times [\exp\left(\hat{\beta }\right)-1]$. $q_{pred}$ is FDR-adjusted within each predictor family across the ten outcomes; $q_{global}$ is FDR-adjusted across all coefficients in this table. Table S3. Median quantile-regression sensitivity findings with predictor-wise FDR q<0.05. Full quantile-regression output is available from the analysis scripts. Table S4. Fisher exact-test findings among low-detection elements with predictor-wise FDR q<0.05. Tests were applied to detection status, not concentration. Table S5. Spearman correlation matrix for log-transformed shifted concentrations of the ten adult primary elements. Asterisks indicate FDR q<0.05. Table S6. Ward hierarchical clustering diagnostics for raw and residualized adult element profiles. Silhouette is the average silhouette width. Table S7. Raw Ward k=2 participant-cluster description. Element medians are in μg/g dry weight. Table S8. Raw Ward k=3 sensitivity description. The four-participant cluster is interpreted as a sentinel pattern rather than a stable exposure class. Element medians are in μg/g dry weight. Table S9. Residualized Ward k=2 participant-cluster description after adjustment for sex, age, hair treatments, cosmetic products and sample mass. Element medians are raw concentrations in μg/g dry weight. Table S10. Gaussian mixture-model sensitivity analysis. Lower BIC values indicate relatively better fit within each input matrix. Table S11. Territorial summaries used for the exploratory spatial figure. Concentration medians are reported in μg/g dry weight; detection summaries are reported as percentages. Table S12. Quality-assurance fields recommended for extreme values and analytically sensitive observations. Table S13. Laboratory LOD and LOQ values used where applicable in the low-detection sensitivity analyses. Units are μg/g. Table S14. Analytical-reporting fields and availability in the statistical archive used for revision. Table S15. Exploratory global tests for territorial differences. Kruskal–Wallis tests were applied to concentration medians; contingency-table tests were applied to detection frequencies. Table S16. Bootstrap stability of Sorrento Peninsula concentration medians. Values are in μg/g. Bootstrap intervals are percentile 95% confidence intervals based on 5000 resamples. Table S17. Zone-specific detection frequencies for low-detection elements included in the spatial display. Values are percentages. Table S18. Low-detection sensitivity analysis for As, Cd and V using laboratory LOD values. Only associations with FDR-adjusted q<0.05 in any sensitivity scheme are shown. No As or Cd association met this threshold.
