# Supplementary material for: Hair Metal Concentrations in Adults Living in Campania, Italy: A Cross-Sectional Biomonitoring Study in a Mixed Geogenic–Anthropogenic Exposure Setting
Source: Toxics. 2026 Aug 27;14(9):768. doi: 10.3390/toxics14090768 (PMC13611674; doi:10.3390/toxics14090768)
Supplement: Supplementary file 1 [file toxics-14-00768-s001.zip › toxics-4468837-supplementary.pdf]

Article

Supplementary Materials: Hair Metal Concentrations in Adults Living in Campania, Italy: Adult Cross-Sectional Biomonitoring in a Mixed Geogenic–Anthropogenic Exposure Setting

Angelo Riccio, Juri Rimauro, Silvio Del Pizzo, Elena Chianese and Rossella Farina

Supplementary Materials

Continuous models were restricted to Al, Ba, Ca, Cr, Cu, K, Mg, Pb, Se and Zn because these elements were detected in at least 70% of adults. Fe was below the threshold at 69.4% and was handled as a low-detection element. Supplementary Tables S6–S10 report the exploratory clustering analysis. Supplementary Tables S13–S17 report the additional LOD/LOQ, analytical-reporting, spatial-testing, bootstrap-stability and low-detection sensitivity analyses added during revision.

Supplementary Table S11 reports the territorial summaries used for the robust exploratory spatial figure in the main m anuscript. For Al, Cr, Fe, Mn, Pb and Zn, the mapped statistic is the zone-specific median c oncentration. For As, Cd and V, the mapped statistic is the zone-specific detection frequency, because these elements were below the 70% detection threshold. Supplementary Table S12 provides a transparent quality-assurance reporting framework for extreme values and analytically sensitive observations, and Table S13 reports the laboratory LOD/LOQ values supplied during revision.

Table S1. Arithmetic means of hair element concentrations in Campanian adults ($n = 134$). Units: µg/g dry weight. Means are provided for completeness but are not primary summaries for right-skewed elements.

Element	n	Mean	SD	Min	Max
Ag	134	1.40	6.87	0.000	75.89
Al	134	3.30	5.43	0.000	37.65
As	134	0.019	0.143	0.000	1.56
B	134	1.30	6.61	0.000	74.30
Ba	134	1.16	1.85	0.000	11.77
Be	134	0.009	0.097	0.000	1.12
Bi	134	0.013	0.090	0.000	0.893
Ca	134	1961.5	1912.0	0.000	9614.6
Cd	134	3.52	27.27	0.000	311.2
Co	134	0.041	0.115	0.000	0.893
Cr	134	0.910	0.944	0.000	10.49
Cu	134	19.22	15.78	0.000	81.75
Fe	134	6.34	12.35	0.000	92.96
K	134	28.26	41.84	0.000	311.4
Mg	134	138.8	136.2	0.000	763.5
Mn	134	0.398	0.689	0.000	4.71

Element	n	Mean	SD	Min	Max
Mo	134	0.229	1.59	0.000	18.36
Ni	134	0.170	0.442	0.000	3.91
Pb	134	13.68	83.71	0.000	887.1
Se	134	1.47	9.18	0.000	106.3
Tl	134	0.000	0.000	0.000	0.000
U	134	0.022	0.078	0.000	0.530
V	134	0.084	0.121	0.000	0.503
Zn	134	169.1	107.4	0.000	1008.1

Table S2. Full HC3-robust multivariable regression output for the ten primary adult elements. Outcomes are log-transformed concentrations with an element-specific offset. Percentage changes are $100 \times [\exp(\beta) - 1]$. q^{pred} is FDR-adjusted within each predictor family across the ten outcomes; q^{global} is FDR-adjusted across all coefficients in this table.

Predictor	Element	$\hat{\beta}$	95% CI (log)	Δ%	95% CI (%)	p	q_{pred}	q_{global}
Male sex	Al	-0.359	[-0.988, 0.270]	-30.2	[-62.8, 31.0]	0.263	0.439	0.777
Fish: 1–3/week	Al	-0.033	[-0.571, 0.504]	-3.3	[-43.5, 65.6]	0.903	0.924	0.957
Fish: > 3/week	Al	-0.314	[-1.532, 0.905]	-26.9	[-78.4, 147.2]	0.614	0.764	0.957
Current smoker	Al	0.103	[-0.558, 0.765]	10.9	[-42.8, 114.8]	0.759	0.979	0.957
Passive smoke exposure	Al	0.185	[-0.435, 0.806]	20.4	[-35.3, 123.8]	0.558	0.884	0.957
Hair treatments	Al	0.574	[-0.079, 1.228]	77.6	[-7.6, 241.4]	0.085	0.425	0.526
Cosmetic products	Al	-0.065	[-0.738, 0.609]	-6.3	[-52.2, 83.9]	0.851	0.934	0.957
Tap-water use	Al	-0.068	[-0.637, 0.500]	-6.6	[-47.1, 64.8]	0.814	0.906	0.957
Medication use	Al	-0.035	[-0.623, 0.552]	-3.5	[-46.4, 73.7]	0.906	0.906	0.957
Supplement use	Al	0.448	[-0.133, 1.030]	56.6	[-12.5, 180.0]	0.131	0.559	0.636
Occupational/environmental risk	Al	-0.244	[-0.889, 0.400]	-21.7	[-58.9, 49.2]	0.458	0.658	0.955
Age, per year	Al	0.011	[-0.008, 0.030]	1.1	[-0.8, 3.1]	0.249	0.312	0.772
Sample mass, per g	Al	-2.310	[-5.363, 0.743]	-90.1	[-99.5, 110.3]	0.138	0.23	0.636
Male sex	Ba	0.271	[-0.424, 0.965]	31.1	[-34.5, 162.5]	0.445	0.542	0.948
Fish: 1–3/week	Ba	-0.032	[-0.702, 0.637]	-3.2	[-50.4, 89.1]	0.924	0.924	0.957
Fish: > 3/week	Ba	-0.266	[-1.445, 0.913]	-23.4	[-76.4, 149.2]	0.658	0.764	0.957
Current smoker	Ba	0.319	[-0.314, 0.952]	37.6	[-26.9, 159.1]	0.323	0.979	0.798
Passive smoke exposure	Ba	0.285	[-0.376, 0.947]	33.0	[-31.3, 157.7]	0.398	0.884	0.884
Hair treatments	Ba	0.036	[-0.740, 0.813]	3.7	[-52.3, 125.4]	0.927	0.927	0.957
Cosmetic products	Ba	0.104	[-0.679, 0.887]	10.9	[-49.3, 142.7]	0.795	0.934	0.957
Tap-water use	Ba	0.286	[-0.307, 0.878]	33.1	[-26.4, 140.7]	0.344	0.906	0.814
Medication use	Ba	0.071	[-0.563, 0.705]	7.3	[-43.0, 102.3]	0.826	0.906	0.957
Supplement use	Ba	0.005	[-0.570, 0.580]	0.5	[-43.4, 78.5]	0.987	0.987	0.987
Occupational/environmental risk	Ba	0.177	[-0.470, 0.824]	19.3	[-37.5, 128.0]	0.592	0.658	0.957

Predictor	Element	$\hat{\beta}$	95% CI (log)	Δ%	95% CI (%)	p	q_{pred}	q_{global}
Age, per year	Ba	-0.004	[-0.026, 0.017]	-0.4	[-2.5, 1.7]	0.683	0.683	0.957
Sample mass, per g	Ba	-4.569	[-12.476, 3.337]	-99.0	[-100.0, 2714.4]	0.257	0.322	0.777
Male sex	Ca	-0.957	[-1.313, -0.601]	-61.6	[-73.1, -45.2]	1.35e-07	1.35e-06	8.81e-06
Fish: 1–3/week	Ca	0.213	[-0.131, 0.557]	23.7	[-12.3, 74.5]	0.226	0.924	0.733
Fish: > 3/week	Ca	0.316	[-0.172, 0.805]	37.2	[-15.8, 123.6]	0.204	0.764	0.698
Current smoker	Ca	-0.043	[-0.414, 0.329]	-4.2	[-33.9, 39.0]	0.823	0.979	0.957
Passive smoke exposure	Ca	-0.048	[-0.415, 0.318]	-4.7	[-34.0, 37.4]	0.795	0.884	0.957
Hair treatments	Ca	0.421	[-0.000, 0.843]	52.4	[-0.0, 132.3]	0.0502	0.425	0.428
Cosmetic products	Ca	0.299	[-0.086, 0.684]	34.8	[-8.3, 98.1]	0.128	0.321	0.636
Tap-water use	Ca	0.103	[-0.198, 0.403]	10.8	[-18.0, 49.7]	0.504	0.906	0.957
Medication use	Ca	-0.242	[-0.605, 0.121]	-21.5	[-45.4, 12.8]	0.191	0.635	0.698
Supplement use	Ca	-0.187	[-0.474, 0.100]	-17.1	[-37.7, 10.5]	0.201	0.559	0.698
Occupational/environmental risk	Ca	0.109	[-0.218, 0.435]	11.5	[-19.6, 54.4]	0.514	0.658	0.957
Age, per year	Ca	0.008	[-0.003, 0.018]	0.8	[-0.3, 1.9]	0.146	0.243	0.636
Sample mass, per g	Ca	-1.889	[-3.854, 0.075]	-84.9	[-97.9, 7.8]	0.0595	0.149	0.43
Male sex	Cr	-0.401	[-0.684, -0.118]	-33.0	[-49.5, -11.2]	0.00544	0.0115	0.106
Fish: 1–3/week	Cr	0.065	[-0.252, 0.382]	6.7	[-22.3, 46.5]	0.69	0.924	0.957
Fish: > 3/week	Cr	0.217	[-0.326, 0.760]	24.3	[-27.8, 113.9]	0.433	0.764	0.937
Current smoker	Cr	0.162	[-0.149, 0.474]	17.6	[-13.9, 60.6]	0.307	0.979	0.798
Passive smoke exposure	Cr	-0.087	[-0.443, 0.269]	-8.3	[-35.8, 30.9]	0.633	0.884	0.957
Hair treatments	Cr	-0.082	[-0.336, 0.171]	-7.9	[-28.5, 18.7]	0.526	0.927	0.957
Cosmetic products	Cr	0.267	[-0.035, 0.570]	30.6	[-3.5, 76.8]	0.0834	0.278	0.526
Tap-water use	Cr	0.018	[-0.276, 0.311]	1.8	[-24.1, 36.4]	0.906	0.906	0.957
Medication use	Cr	-0.083	[-0.472, 0.305]	-8.0	[-37.6, 35.6]	0.673	0.906	0.957
Supplement use	Cr	0.023	[-0.243, 0.288]	2.3	[-21.5, 33.4]	0.866	0.987	0.957
Occupational/environmental risk	Cr	-0.300	[-0.605, 0.004]	-25.9	[-45.4, 0.4]	0.0529	0.529	0.428

Predictor	Element	$\hat{\beta}$	95% CI (log)	Δ%	95% CI (%)	p	q_{pred}	q_{global}
Age, per year	Cr	0.007	[-0.007, 0.021]	0.7	[-0.7, 2.2]	0.307	0.341	0.798
Sample mass, per g	Cr	-1.923	[-4.252, 0.407]	-85.4	[-98.6, 50.2]	0.106	0.212	0.573
Male sex	Cu	-0.473	[-0.753, -0.192]	-37.7	[-52.9, -17.5]	0.000956	0.00319	0.0249
Fish: 1–3/week	Cu	0.117	[-0.142, 0.376]	12.4	[-13.2, 45.7]	0.376	0.924	0.872
Fish: $>$ 3/week	Cu	0.104	[-0.403, 0.612]	11.0	[-33.2, 84.3]	0.687	0.764	0.957
Current smoker	Cu	-0.018	[-0.326, 0.291]	-1.8	[-27.9, 33.8]	0.91	0.979	0.957
Passive smoke exposure	Cu	-0.156	[-0.439, 0.128]	-14.4	[-35.6, 13.6]	0.282	0.884	0.798
Hair treatments	Cu	0.123	[-0.211, 0.457]	13.1	[-19.0, 58.0]	0.47	0.927	0.955
Cosmetic products	Cu	0.353	[0.043, 0.663]	42.3	[4.4, 94.1]	0.0256	0.233	0.416
Tap-water use	Cu	-0.046	[-0.302, 0.209]	-4.5	[-26.1, 23.3]	0.722	0.906	0.957
Medication use	Cu	-0.330	[-0.642, -0.019]	-28.1	[-47.4, -1.9]	0.0375	0.375	0.428
Supplement use	Cu	-0.030	[-0.254, 0.194]	-2.9	[-22.4, 21.4]	0.794	0.987	0.957
Occupational/environmental risk	Cu	0.176	[-0.073, 0.425]	19.3	[-7.0, 52.9]	0.165	0.658	0.692
Age, per year	Cu	0.007	[-0.002, 0.016]	0.7	[-0.2, 1.7]	0.139	0.243	0.636
Sample mass, per g	Cu	-2.229	[-4.443, -0.015]	-89.2	[-98.8, -1.5]	0.0485	0.149	0.428
Male sex	K	0.318	[-0.338, 0.974]	37.4	[-28.7, 164.8]	0.343	0.489	0.814
Fish: 1–3/week	K	-0.139	[-0.690, 0.413]	-12.9	[-49.9, 51.2]	0.623	0.924	0.957
Fish: $>$ 3/week	K	-0.230	[-1.049, 0.589]	-20.5	[-65.0, 80.3]	0.582	0.764	0.957
Current smoker	K	0.384	[-0.178, 0.946]	46.8	[-16.3, 157.5]	0.18	0.979	0.698
Passive smoke exposure	K	0.227	[-0.384, 0.839]	25.5	[-31.9, 131.4]	0.466	0.884	0.955
Hair treatments	K	0.070	[-0.578, 0.718]	7.2	[-43.9, 104.9]	0.833	0.927	0.957
Cosmetic products	K	0.062	[-0.591, 0.716]	6.4	[-44.6, 104.5]	0.852	0.934	0.957
Tap-water use	K	0.046	[-0.415, 0.507]	4.7	[-34.0, 66.0]	0.846	0.906	0.957
Medication use	K	0.053	[-0.493, 0.599]	5.4	[-38.9, 82.0]	0.849	0.906	0.957
Supplement use	K	0.091	[-0.390, 0.573]	9.6	[-32.3, 77.3]	0.71	0.987	0.957
Occupational/environmental risk	K	0.155	[-0.386, 0.696]	16.8	[-32.0, 100.6]	0.574	0.658	0.957

Predictor	Element	$\hat{\beta}$	95% CI (log)	Δ%	95% CI (%)	p	q_{pred}	q_{global}
Age, per year	K	0.021	[0.002, 0.040]	2.1	[0.2, 4.0]	0.0292	0.148	0.421
Sample mass, per g	K	-4.954	[-14.723, 4.814]	-99.3	[-100.0, 12227.4]	0.32	0.356	0.798
Male sex	Mg	-0.738	[-1.121, -0.356]	-52.2	[-67.4, -29.9]	0.000154	0.000769	0.005
Fish: 1–3/week	Mg	0.214	[-0.212, 0.641]	23.9	[-19.1, 89.8]	0.325	0.924	0.798
Fish: > 3/week	Mg	0.190	[-0.375, 0.756]	21.0	[-31.3, 112.9]	0.509	0.764	0.957
Current smoker	Mg	0.006	[-0.417, 0.429]	0.6	[-34.1, 53.5]	0.979	0.979	0.986
Passive smoke exposure	Mg	0.020	[-0.376, 0.416]	2.0	[-31.3, 51.6]	0.921	0.921	0.957
Hair treatments	Mg	0.291	[-0.143, 0.725]	33.7	[-13.4, 106.4]	0.19	0.632	0.698
Cosmetic products	Mg	0.071	[-0.407, 0.548]	7.3	[-33.4, 73.1]	0.771	0.934	0.957
Tap-water use	Mg	0.095	[-0.226, 0.415]	9.9	[-20.2, 51.5]	0.563	0.906	0.957
Medication use	Mg	-0.217	[-0.620, 0.186]	-19.5	[-46.2, 20.5]	0.291	0.728	0.798
Supplement use	Mg	-0.007	[-0.342, 0.329]	-0.7	[-29.0, 38.9]	0.969	0.987	0.984
Occupational/environmental risk	Mg	0.136	[-0.240, 0.511]	14.5	[-21.4, 66.8]	0.48	0.658	0.957
Age, per year	Mg	0.013	[0.001, 0.025]	1.3	[0.1, 2.5]	0.041	0.148	0.428
Sample mass, per g	Mg	-3.035	[-4.547, -1.524]	-95.2	[-98.9, -78.2]	8.3e-05	0.000415	0.00359
Male sex	Pb	0.127	[-0.622, 0.876]	13.6	[-46.3, 140.2]	0.74	0.74	0.957
Fish: 1–3/week	Pb	-0.154	[-0.858, 0.550]	-14.3	[-57.6, 73.3]	0.668	0.924	0.957
Fish: > 3/week	Pb	-0.659	[-1.873, 0.554]	-48.3	[-84.6, 74.1]	0.287	0.764	0.798
Current smoker	Pb	0.148	[-0.556, 0.851]	15.9	[-42.6, 134.3]	0.681	0.979	0.957
Passive smoke exposure	Pb	0.117	[-0.645, 0.878]	12.4	[-47.5, 140.6]	0.764	0.884	0.957
Hair treatments	Pb	-0.090	[-0.834, 0.653]	-8.7	[-56.6, 92.1]	0.811	0.927	0.957
Cosmetic products	Pb	0.034	[-0.771, 0.839]	3.4	[-53.8, 131.4]	0.934	0.934	0.957
Tap-water use	Pb	0.085	[-0.552, 0.723]	8.9	[-42.4, 106.1]	0.793	0.906	0.957
Medication use	Pb	-0.136	[-0.920, 0.647]	-12.8	[-60.1, 90.9]	0.733	0.906	0.957
Supplement use	Pb	-0.163	[-0.790, 0.465]	-15.0	[-54.6, 59.1]	0.611	0.987	0.957
Occupational/environmental risk	Pb	0.288	[-0.384, 0.959]	33.3	[-31.9, 161.0]	0.401	0.658	0.884

Predictor	Element	$\hat{\beta}$	95% CI (log)	Δ%	95% CI (%)	p	q_{pred}	q_{global}
Age, per year	Pb	0.022	[-0.004, 0.048]	2.2	[-0.4, 4.9]	0.103	0.243	0.573
Sample mass, per g	Pb	-4.190	[-10.469, 2.089]	-98.5	[-100.0, 707.6]	0.191	0.273	0.698
Male sex	Se	-0.188	[-0.721, 0.344]	-17.2	[-51.4, 41.1]	0.488	0.542	0.957
Fish: 1–3/week	Se	-0.488	[-1.027, 0.051]	-38.6	[-64.2, 5.2]	0.076	0.76	0.52
Fish: $>$ 3/week	Se	-0.631	[-1.702, 0.439]	-46.8	[-81.8, 55.2]	0.248	0.764	0.772
Current smoker	Se	0.292	[-0.379, 0.963]	33.9	[-31.5, 162.0]	0.394	0.979	0.884
Passive smoke exposure	Se	-0.157	[-0.718, 0.403]	-14.6	[-51.2, 49.6]	0.582	0.884	0.957
Hair treatments	Se	-0.038	[-0.583, 0.508]	-3.7	[-44.2, 66.1]	0.892	0.927	0.957
Cosmetic products	Se	0.137	[-0.453, 0.726]	14.6	[-36.4, 106.7]	0.65	0.934	0.957
Tap-water use	Se	-0.167	[-0.741, 0.406]	-15.4	[-52.3, 50.1]	0.568	0.906	0.957
Medication use	Se	-0.542	[-1.187, 0.103]	-41.9	[-69.5, 10.8]	0.0994	0.497	0.573
Supplement use	Se	0.492	[-0.013, 0.996]	63.5	[-1.2, 170.8]	0.056	0.559	0.428
Occupational/environmental risk	Se	-0.115	[-0.655, 0.425]	-10.8	[-48.0, 53.0]	0.677	0.677	0.957
Age, per year	Se	0.013	[-0.007, 0.033]	1.3	[-0.7, 3.4]	0.204	0.291	0.698
Sample mass, per g	Se	-1.129	[-5.983, 3.725]	-67.7	[-99.7, 4046.0]	0.648	0.648	0.957
Male sex	Zn	-0.274	[-0.468, -0.080]	-23.9	[-37.4, -7.6]	0.00573	0.0115	0.106
Fish: 1–3/week	Zn	-0.017	[-0.254, 0.219]	-1.7	[-22.4, 24.5]	0.886	0.924	0.957
Fish: $>$ 3/week	Zn	-0.020	[-0.377, 0.337]	-2.0	[-31.4, 40.0]	0.911	0.911	0.957
Current smoker	Zn	-0.076	[-0.333, 0.180]	-7.3	[-28.3, 19.7]	0.56	0.979	0.957
Passive smoke exposure	Zn	-0.176	[-0.414, 0.062]	-16.1	[-33.9, 6.4]	0.147	0.884	0.636
Hair treatments	Zn	0.029	[-0.155, 0.213]	2.9	[-14.4, 23.7]	0.758	0.927	0.957
Cosmetic products	Zn	0.230	[0.003, 0.456]	25.8	[0.3, 57.7]	0.0466	0.233	0.428
Tap-water use	Zn	0.037	[-0.162, 0.236]	3.8	[-14.9, 26.6]	0.715	0.906	0.957
Medication use	Zn	-0.020	[-0.297, 0.257]	-1.9	[-25.7, 29.3]	0.889	0.906	0.957
Supplement use	Zn	0.116	[-0.071, 0.303]	12.3	[-6.8, 35.4]	0.223	0.559	0.733
Occupational/environmental risk	Zn	0.102	[-0.101, 0.304]	10.7	[-9.6, 35.5]	0.326	0.658	0.798

Predictor	Element	$\hat{\beta}$	95% CI (log)	Δ%	95% CI (%)	p	q_{pred}	q_{global}
Age, per year	Zn	0.007	[0.000, 0.015]	0.8	[0.0, 1.5]	0.0445	0.148	0.428
Sample mass, per g	Zn	-2.638	[-3.441, -1.836]	-92.9	[-96.8, -84.1]	1.15e-10	1.15e-09	1.49e-08

Table S3. Median quantile-regression sensitivity findings with predictor-wise FDR $q < 0.05$. Full quantile-regression output is available from the analysis scripts.

Predictor	Element	$\hat{\beta}$	p	q
Male sex	Ca	-1.196	1.63e-10	1.63e-09
Male sex	Cu	-0.347	0.0021	0.00701
Male sex	Mg	-0.997	5.11e-05	0.000255
Age, per year	Cr	0.012	0.00114	0.0114
Sample mass, per g	Ba	-4.492	0.0261	0.029
Sample mass, per g	Ca	-2.439	0.00688	0.00983
Sample mass, per g	Cr	-2.164	5.2e-05	0.000173
Sample mass, per g	Cu	-2.285	0.000114	0.000228
Sample mass, per g	K	-8.640	3.29e-07	1.65e-06
Sample mass, per g	Mg	-2.960	0.0177	0.0221
Sample mass, per g	Pb	-5.682	8.91e-05	0.000223
Sample mass, per g	Se	-2.781	0.00554	0.00924
Sample mass, per g	Zn	-2.777	2.79e-11	2.79e-10

Table S4. Fisher exact-test findings among low-detection elements with predictor-wise FDR $q < 0.05$. Tests were applied to detection status, not concentration.

Predictor	Element	p	q
Hair treatments	Co	0.000128	0.0018
Hair treatments	Mn	0.00096	0.00672
Hair treatments	Ni	0.00596	0.0208
Hair treatments	V	0.00163	0.0076
Male sex	Ag	2.99e-07	4.18e-06
Male sex	Co	0.000988	0.00346
Male sex	Mn	3.52e-06	2.47e-05
Male sex	Ni	0.0117	0.0327
Male sex	V	0.000364	0.0017

Table S5. Spearman correlation matrix for log-transformed shifted concentrations of the ten adult primary elements. Asterisks indicate FDR $q < 0.05$.

	Al	Ba	Ca	Cr	Cu	K	Mg	Pb	Se	Zn
Al	1.00	0.37*	0.32*	0.53*	0.42*	0.35*	0.38*	0.46*	0.35*	0.35*
Ba	0.37*	1.00	0.31*	0.16	0.30*	0.68*	0.46*	0.55*	0.34*	0.40*
Ca	0.32*	0.31*	1.00	0.32*	0.61*	0.12	0.76*	0.25*	0.07	0.41*
Cr	0.53*	0.16	0.32*	1.00	0.45*	0.33*	0.35*	0.34*	0.50*	0.49*
Cu	0.42*	0.30*	0.61*	0.45*	1.00	0.22*	0.50*	0.36*	0.34*	0.48*
K	0.35*	0.68*	0.12	0.33*	0.22*	1.00	0.45*	0.50*	0.41*	0.48*
Mg	0.38*	0.46*	0.76*	0.35*	0.50*	0.45*	1.00	0.39*	0.21*	0.56*
Pb	0.46*	0.55*	0.25*	0.34*	0.36*	0.50*	0.39*	1.00	0.31*	0.37*
Se	0.35*	0.34*	0.07	0.50*	0.34*	0.41*	0.21*	0.31*	1.00	0.51*
Zn	0.35*	0.40*	0.41*	0.49*	0.48*	0.48*	0.56*	0.37*	0.51*	1.00

Exploratory clustering

The clustering analysis was added as an exploratory description of recurrent multi-element profiles. It should not be interpreted as source apportionment. Input variables were the ten elements detected in at least 70% of adults: Al, Ba, Ca, Cr, Cu, K, Mg, Pb, Se and Zn. Concentrations were transformed as $\log(x + o_e)$, winsorized at the 1st and 99th percentiles, and robust-standardized by median and interquartile range. A residualized

version was also analysed after adjustment for sex, age, hair treatments, cosmetic product use and sample mass.

Table S6. Ward hierarchical clustering diagnostics for raw and residualized adult element profiles. Silhouette is the average silhouette width.

Input	k	Silhouette	Minimum cluster n	Cluster sizes
raw	2	0.377	29	1: 105, 2: 29
raw	3	0.378	4	1: 105, 2: 4, 3: 25
raw	4	0.165	4	1: 39, 2: 66, 3: 4, 4: 25
raw	5	0.181	4	1: 39, 2: 66, 3: 4, 4: 11, 5: 14
residual	2	0.183	60	1: 60, 2: 74
residual	3	0.170	23	1: 60, 2: 51, 3: 23
residual	4	0.189	4	1: 60, 2: 51, 3: 4, 4: 19
residual	5	0.191	3	1: 60, 2: 51, 3: 4, 4: 3, 5: 16

Table S7. Raw Ward $k = 2$ participant-cluster description. Element medians are in μg/g dry weight.

Cluster	n	Male (%)	Age median	Mass median (g)	Ca	Mg	Cu	Zn	Pb	Ba	Risk (%)
1	105	45.7	33.0	0.113	1410.0	117.5	16.5	161.3	0.83	0.79	37.1
2	29	65.5	25.0	0.085	640.3	41.5	8.8	99.4	0.42	0.49	48.3

Table S8. Raw Ward $k = 3$ sensitivity description. The four-participant cluster is interpreted as a sentinel pattern rather than a stable exposure class. Element medians are in μg/g dry weight.

Cluster	n	Male (%)	Age median	Mass median (g)	Ca	Mg	Cu	Zn	Pb	Ba	Risk (%)
1	105	45.7	33.0	0.113	1410.0	117.5	16.5	161.3	0.83	0.79	37.1
2	4	100.0	35.0	0.014	974.8	120.9	21.1	224.7	61.68	2.56	100.0
3	25	60.0	25.0	0.095	541.4	35.6	8.1	76.7	0.32	0.26	40.0

Table S9. Residualized Ward $k = 2$ participant-cluster description after adjustment for sex, age, hair treatments, cosmetic products and sample mass. Element medians are raw concentrations in μg/g dry weight.

Cluster	n	Male (%)	Age median	Mass median (g)	Ca	Mg	Cu	Zn	Pb	Ba	Risk (%)
1	60	48.3	31.5	0.100	1903.9	154.9	18.5	178.0	1.46	1.13	46.7
2	74	51.4	30.5	0.111	1029.3	54.3	11.8	137.5	0.49	0.36	33.8

Table S10. Gaussian mixture-model sensitivity analysis. Lower BIC values indicate relatively better fit within each input matrix.

Input	Covariance	k	BIC	AIC
raw	full	4	2841.1	2079.0
raw	full	3	2901.1	2330.3
raw	diag	4	2994.2	2753.7
raw	full	5	3006.7	2053.4
raw	full	2	3020.2	2640.6
residual	full	1	2996.5	2808.1
residual	diag	4	3003.0	2762.5
residual	diag	5	3011.3	2709.9
residual	diag	3	3048.7	2869.0
residual	full	2	3131.2	2751.6

Exploratory spatial summaries

Table S11 reports the zone-level quantities used to generate the revised spatial visualization in the main manuscript. Medians were used for elements with sufficient detection and strongly skewed concentration distributions, whereas detection frequencies were used

for As, Cd and V. This choice avoids implying precision for elements dominated by non-detects and reduces the influence of extreme observations on area-level displays. The Sorrento Peninsula group had the smallest number of adults ($n = 9$) and is therefore interpreted as a descriptive stratum rather than a stable territorial estimate.

Table S11. Territorial summaries used for the exploratory spatial figure. Concentration medians are reported in µg/g dry weight; detection summaries are reported as percentages.

Element	Metric	Naples	North Naples	South Naples	Sorrento Peninsula
Al	Median (µg/g)	2.93	1.21	2.03	3.32
As	Detection (%)	0.0	2.6	3.3	22.2
Cd	Detection (%)	15.6	15.8	23.3	55.6
Cr	Median (µg/g)	0.78	0.76	0.86	1.08
Fe	Median (µg/g)	3.02	2.16	5.08	6.35
Mn	Median (µg/g)	0.35	0.11	0.30	0.24
Pb	Median (µg/g)	0.92	0.80	0.79	1.41
V	Detection (%)	28.1	36.8	60.0	55.6
Zn	Median (µg/g)	165.65	142.58	145.53	194.98

Quality-assurance reporting framework

The statistical dataset identified extreme right-tail values for Pb, Cd, Se and Ag, and the regression and clustering analyses also highlighted sample mass as an analytically relevant covariate. These observations were retained in the adult analysis because they represent either plausible sentinel observations or analytically important records requiring traceable quality assurance. Table S12 lists the quality-control fields that should accompany the final laboratory archive and any future data release. This table is not intended to remove observations from the analysis; rather, it documents the audit trail needed to distinguish true high-burden observations, external contamination and possible analytical artefacts.

Table S12. Quality-assurance fields recommended for extreme values and analytically sensitive observations.

Field	Purpose	Reporting recommendation
Sample identifier audit	Confirms that the sample, questionnaire record and analytical result correspond to the same adult participant.	Retain in laboratory records; public data should use de-identified codes only.
Batch membership	Evaluates whether extreme values cluster in a specific digestion or instrument batch.	Report batch-level checks in the laboratory QA file.
Procedural blanks	Assesses contamination introduced during preparation or digestion.	Record blank results for each batch and flag values close to blank-derived limits.
Calibration range and dilution factor	Checks whether the measured digest concentration fell within the validated analytical range.	Document re-dilution or re-analysis for values exceeding the calibration range.
LOD and LOQ by element	Supports interpretation of non-detects and low-detection elements.	Reported for available elements in Table S13 and retained in the laboratory archive.
CRM or spike recovery	Evaluates analytical accuracy in a matrix or matrix-like material.	Report recovery ranges by element and batch where available.
Duplicate digestion or measurement	Evaluates within-laboratory reproducibility.	Report relative percent difference or relative standard deviation for replicated samples.
Hair mass	Evaluates potential mass-related effects in concentration estimates and detection limits.	Retain the weighed mass for each sample; interpret very low masses cautiously.
Hair treatment and cosmetic status	Helps distinguish endogenous incorporation from possible external or cosmetic contamination.	Retain in the analysis dataset and consider in sensitivity analyses.
Residence and occupation notes	Supports follow-up for sentinel Pb, Cd, Se or Ag observations.	Restrict public release to aggregated geography to prevent re-identification.

Table S13. Laboratory LOD and LOQ values used where applicable in the low-detection sensitivity analyses. Units are $\mu g/g$.

Element	LOQ	LOD
Al	1	0.3
As	0.01	0.003
B	0.5	0.15
Ba	0.1	0.03
Be	0.01	0.003
Bi	0.01	0.003
Ca	50	15
Cd	0.01	0.003
Co	0.1	0.03
Cr	0.01	0.003
Cu	0.1	0.03
Fe	5	1.5
K	150	45.0
Mg	5	1.5
Mn	0.1	0.03
Mo	0.01	0.003
Pb	0.01	0.003
Tl	0.01	0.003
V	0.01	0.003
Zn	0.2	0.06

Additional analytical and statistical checks added during revision

Table S14 summarizes the analytical-reporting fields requested for reproducibility. The available statistical archive contained final concentrations, questionnaire variables, hair masses and the LOD/LOQ values shown in Table S13; not all batch-level instrumental records were embedded in that archive.

Table S14. Analytical-reporting fields and availability in the statistical archive used for revision.

Field	Status in revision archive	Interpretation for the manuscript
ICP–MS platform and acquisition settings	Not available in the statistical archive	Reported as a transparency limitation; not inferred retrospectively.
Element-specific calibration ranges	Not available in the statistical archive	Full analytical validation should be retained in laboratory QA records.
Element-specific LOD/LOQ	Available for 20 elements in the supplied LOD/LOQ workbook	Used directly for the As, Cd and V sensitivity analyses; remaining missing values were not reconstructed.
Procedural blanks	Reported qualitatively as part of the laboratory workflow	Used to justify post-analytical audit language, but not to calculate element-specific LOD/LOQ in this revision.
Replicate digestions	Reported qualitatively for a subset of samples	Supports reproducibility screening; replicate RSDs should be archived in future datasets.
Hair-matrix CRM	Not available for this dataset	Added as a limitation and as a priority for future work.
Spike-recovery data	Not available in the statistical archive	Added as a limitation; future studies should report recoveries by element and batch.
Sample-mass gradient experiment	Not performed	The observed mass association is interpreted as an analytical signal requiring follow-up, not as a biological effect.

Tables S14 and S15 provide the additional quantitative checks requested for the spatial analysis. These tests were exploratory because the study was not spatially balanced by design and because the Sorrento Peninsula stratum included only nine adults.

Table S15. Exploratory global tests for territorial differences. Kruskal–Wallis tests were applied to concentration medians; contingency-table tests were applied to detection frequencies.

Element	Statistic	Global test	p-value	Interpretation
Al	Median concentration	Kruskal–Wallis $H = 3.33$	0.344	No global difference after exploratory testing
Cr	Median concentration	Kruskal–Wallis $H = 7.35$	0.062	Borderline heterogeneity only
Fe	Median concentration	Kruskal–Wallis $H = 6.85$	0.077	Borderline heterogeneity only
Mn	Median concentration	Kruskal–Wallis $H = 4.57$	0.206	No global difference after exploratory testing
Pb	Median concentration	Kruskal–Wallis $H = 0.83$	0.843	No global difference after exploratory testing
Zn	Median concentration	Kruskal–Wallis $H = 9.11$	0.028	Global difference; North Naples vs Sorrento Peninsula remained significant after BH correction
As	Detection frequency	$\chi^2 = 10.11$	0.018	Detection differed across zones
Cd	Detection frequency	$\chi^2 = 7.73$	0.052	Borderline detection heterogeneity
V	Detection frequency	$\chi^2 = 7.60$	0.055	Borderline detection heterogeneity

Table S16. Bootstrap stability of Sorrento Peninsula concentration medians. Values are in µg/g. Bootstrap intervals are percentile 95% confidence intervals based on 5000 resamples.

Element	n	Median	IQR	Bootstrap 95% CI
Al	9	3.32	0.52–10.68	0.33–10.68
Cr	9	1.08	0.95–1.34	0.78–1.41
Fe	9	6.35	3.00–13.79	0.51–27.52
Mn	9	0.24	0.17–0.26	0.00–0.50
Pb	9	1.41	0.48–16.16	0.26–18.70
Zn	9	194.98	176.12–235.94	168.11–279.08

Tables S16 and S17 report the additional checks for low-detection elements. The sensitivity models for As, Cd and V used the laboratory LOD values supplied during revision. These analyses are used to assess robustness, not to replace the detection-frequency strategy in the main analysis.

Table S17. Zone-specific detection frequencies for low-detection elements included in the spatial display. Values are percentages.

Element	Naples	North Naples	South Naples	Sorrento Peninsula	Global p
As	0.0	2.6	3.3	22.2	0.018
Cd	15.6	15.8	23.3	55.6	0.052
V	28.1	36.8	60.0	55.6	0.055

Table S18. Low-detection sensitivity analysis for As, Cd and V using laboratory LOD values. Only associations with FDR-adjusted $q < 0.05$ in any sensitivity scheme are shown. No As or Cd association met this threshold.

Element	Scheme	LOD	Predictor	Coefficient	p-value	q-value
V	LOD-based Tobit	0.003	Male sex	-2.848	0.00233	0.0233
V	LOD-based Tobit	0.003	Age (standardized)	1.137	0.00686	0.0343
